# Content specificity of attentional bias to COVID-19 threat-related information in trait anxiety

**DOI:** 10.3389/fpsyt.2023.1254349

**Published:** 2023-11-16

**Authors:** Yiming Zhao, Xun Jia, Shunjie Pan, Haifeng Ji, Yanmei Wang

**Affiliations:** ^1^Shanghai Key Laboratory of Mental Health and Psychological Crisis Intervention, School of Psychology and Cognitive Science, East China Normal University, Shanghai, China; ^2^Shanghai Changning Mental Health Center, Shanghai, China

**Keywords:** attentional bias, COVID-19, trait anxiety, dot-probe task, content specificity

## Abstract

**Introduction:**

Anxious individuals selectively attend to threatening information, but it remains unclear whether attentional bias can be generalized to traumatic events, such as the COVID-19 pandemic. Previous studies suggested that specific threats related to personal experiences can elicit stronger attentional bias than general threats. The current study aimed to investigate the relationship between content-specific attentional bias and trait anxiety during the COVID-19 pandemic.

**Methods:**

Attentional bias was assessed using the dot-probe task with COVID-19-related, general threat-related, and neutral words at two exposure times, 200 and 500 ms.

**Results:**

We found participants with high trait anxiety exhibited attentional bias toward COVID-19- related stimuli and attentional bias away from general threat-related stimuli, while participants with low trait anxiety showed attentional bias away from both types of stimuli.

**Discussion:**

Results suggest that individuals with high trait anxiety show a content-specific attentional bias to COVID-19-related information during the COVID-19 pandemic. Apart from the innate attentional bias toward biological threats, individuals with high trait anxiety may also learn from trauma and develop trauma-specific attentional bias.

## 1. Introduction

The COVID-19 pandemic has had a profound impact on multiple aspects of life, causing widespread traumatic stress for many people (1). In March 2022, Shanghai was confronted with a recurrence of the COVID-19 pandemic, leading to the adoption of lockdown and quarantine policies in high-risk areas. Citizens in Shanghai faced enormous uncertainty and psychological challenge. The COVID-19 pandemic has been consistently proven to elevate people’s anxiety symptoms (2), but interrelatedly, anxious individuals may also selectively attend to negative information regarding the COVID-19 pandemic (3). This differential attentional allocation toward threatening stimuli compared with neutral stimuli is conceptualized as attentional bias (4, 5). Attentional bias is also considered an underlying mechanism of the development and maintenance of anxiety disorders (6). From an evolutionary perspective, the selective attentional mechanisms toward environmental threats have survival significance (7). However, the persistence of attentional bias toward COVID-19-related negative information may exacerbate anxiety symptoms and interfere with individual’s ability to cope effectively.

Previous studies have suggested that there was a positive association between attentional bias toward COVID-19-related stimuli and anxiety symptoms. For example, Cannito et al. (3) found levels of health anxiety predicted attentional bias toward COVID-19 virus-related objects in the dot-probe task. Similarly, Albery et al. (8) found the attentional bias indices were positively correlated with COVID-19 anxiety syndrome using the same task. However, it remains unclear whether anxious individuals exhibit a stronger attentional bias toward COVID-19-related stimuli compared to general threat-related stimuli after chronic exposure to the COVID-19 pandemic. The relationship between anxiety and COVID-19-related attentional bias can shed light on anxious people’s susceptibility to traumatic events. Apart from the innate attentional bias toward biological threats, they may also learn from trauma and develop a trauma-specific attentional bias. This specific hypervigilance toward potential threats may contribute to the development of anxiety disorders. Thus, exploring attentional bias in the COVID-19 pandemic has implications for research on anxiety disorder mechanisms and for anxiety disorder intervention.

A prior meta-analysis indicates that specific threatening stimuli which is related to individuals’ anxiety type (e.g., faces for social phobia) can elicit a stronger attentional bias than general threat-related stimuli (9). This small but significant effect is not moderated by age, type of anxiety disorder, experimental paradigms, and type of content-incongruent threatening stimuli. Considering that anxiety symptoms are linked to unique patterns of processing personally related threatening information (10), individuals with elevated anxiety symptoms may prioritize and show heightened sensitivity toward specific threats which are related to their experiences or concerns (9). For example, Zinchenko et al. (11) found that individuals with post-traumatic stress disorder (PTSD) who had survived a factory collapse exhibited a content-sensitive dissociation when faced with emotional stimuli. They responded more quickly to emotional buildings than neutral buildings, while they responded more slowly to emotional faces than neutral faces. However, conflicting results regarding specificity have also been reported. For instance, Maidenberg et al. (12) found that participants with panic disorder responded slower to both panic-related and general threat-related words than healthy participants. Thus, further research is needed to explore the specificity in anxiety-linked attentional bias. Moreover, most previous studies have focused on PTSD or a specific anxiety disorder (e.g., social anxiety disorder or spider phobia), while research on specificity in trait anxiety is scarce. Pergamin-Hight et al. (9) have recommended future research to focus on personalized specificity in anxiety disorders where worry is not specific, such as generalized anxiety disorder and trait anxiety. The personalized approach could examine a higher order of content specificity beyond disorder-congruent content and contribute to the optimization of Attention Bias Modification Treatments (ABMT), providing both theoretical and intervention implications.

Individuals with high levels of trait anxiety have been shown to process and react differently to threat-related resources compared to those with low levels of trait anxiety (13). Trait anxiety is associated with exaggeration of the risk of encountering threats and the risk of facing adverse outcomes caused by the threats (14), as well as a memory bias toward threatening information (15). Furthermore, individuals with high levels of trait anxiety have lower cognitive flexibility (16, 17), making it harder for them to adjust their behavioral, emotional, and cognitive responses when facing new information. During the COVID-19 pandemic, individuals high in trait anxiety may allocate more attention to COVID-19-related information, interpret the pandemic as having a catastrophic outcome (e.g., long-term lockdown, shortages of food supplies), exhibit an enhanced memory of negative news related to the COVID-19 pandemic, and experience maladaptation. Chronic exposure to stressful environments may contribute to attentional bias toward threats (18), and the attentional bias after trauma exposure occurs regardless of PTSD (19). Therefore, it is hypothesized that individuals high in trait anxiety will exhibit attentional bias toward both general threat-related stimuli and COVID-19-related stimuli.

COVID-19 stimuli are distinct from ordinary disease stimuli as they have a comprehensive impact on multiple domains of life. As proposed by Taylor (20), the psychological challenges brought about by pandemics are dynamic in nature. Initially, contamination concerns were the primary challenge, but over time, the COVID-19 pandemic has given rise to concerns about unemployment, food shortages, social restrictions, quarantine, and financial issues (21). Thus, it may be hypothesized that attentional bias toward COVID-19-related stimuli is more likely to be associated with anxiety symptoms at a general level (e.g., trait anxiety) rather than being solely linked to health anxiety.

Regarding the temporal mechanisms underlying attentional bias, there has been consistent debate on whether anxiety symptoms are linked with elevated orienting toward, or impaired disengagement from, threatening stimuli. The vigilance-avoidance model and the attention maintenance model emerged as two dominant views. In the vigilance-avoidance model, anxious individuals demonstrate an initial vigilance toward threatening stimuli and a subsequent avoidance away from the stimuli (22). In contrast, in the attention maintenance model, anxious individuals experience difficulty in disengaging from threatening stimuli (23). Both two models were supported by empirical evidence. For example, Mogg and Bradley (24) found attentional vigilance at a shorter exposure time (100 ms) and found subsequent attentional avoidance at a longer exposure time (500 ms) in a sample of non-clinical anxious individuals. Difficulty in disengagement was also invariably found in anxious individuals (25, 26). Although the two models are seemingly incompatible, recent studies have discovered that engagement and disengagement may coexist in attentional bias as independent pathways (27, 28). Individuals may at first exhibit facilitated vigilance toward threat-related stimuli, and then overtly avoid the threat while covertly processing the threatening information (29).

A range of experimental paradigms have been developed to measure attentional bias, including the widely-used dot-probe task. In this task, a neutral stimulus and a threat-related stimulus are presented simultaneously on a computer screen (5). Subsequently, a probe appears in one of the two locations previously occupied by the stimuli, either in the same location as the threat-related stimulus (congruent trial) or in the opposite location (incongruent trial). Participants are instructed to respond as quickly as possible to the probe’s appearance. The dot-probe task has several advantages over other measures of attentional bias. Firstly, it can be used to assess both the direction and magnitude of attentional bias (30). Additionally, the dot-probe task involves competition between two stimuli, which makes it more sensitive to the occurrence of attentional bias than other tasks, such as the spatial cueing task, which only presents one stimulus at a time (4).

A previous meta-analysis suggested that stimuli types (words or pictures) may also be an important factor in the dot-probe task (4). Specifically, it was found that subclinical anxious individuals exhibited attentional bias toward both word and picture stimuli, without significant differences between the two. However, pictures may not be explicit enough to represent abstract concepts related to COVID-19, such as quarantine, fever, and contagion. Words may be more appropriate in the context of the COVID-19 pandemic, as they can remove ambiguity. It was also suggested that word stimuli were more appropriate than picture stimuli when the threatening information was conceptual instead of perceptual (29). Thus, the current study utilized word stimuli.

Due to the inconsistent results in previous COVID-19-related attentional bias studies and the lack of evidence regarding how trait anxiety is associated with COVID-19 attentional bias, further research is needed. According to evolutionary models, all humans possess an innate ability to rapidly detect environmental threats of survival significance, regardless of their susceptibility to anxiety (22). However, it remains unclear whether selective attentional allocation can also be acquired through stressful experiences. The current study aims to explore whether individuals with high trait anxiety are more prone to learn from their stressful experiences and develop exaggerated psychological responses than individuals with low trait anxiety. This content-specific hypervigilance toward potential threats may be attributable to trait anxiety and may contribute to the development of anxiety disorders. Additionally, the current study aims to verify whether individuals with low trait anxiety selectively attend to general threat-related or COVID-19-related stimuli. The current study utilized the dot-probe task to examine the association between trait anxiety and attentional bias toward COVID-19-related and general threat-related word stimuli in two presentation times (200 and 500 ms) during the COVID-19 pandemic. We hypothesize that individuals with high trait anxiety would exhibit attentional bias toward both COVID-19-related and general threat-related stimuli, and the effect of attentional bias toward COVID-19-related stimuli would be stronger. We also hypothesize that individuals with low trait anxiety would not exhibit attentional bias toward COVID-19-related or general threat-related stimuli.

## 2. Materials and methods

### 2.1. Participants

*A priori* power analysis using G*power 3.1.9.7 (31) was performed to estimate the sample size necessary for the interaction effect at 90% power. The effect size was set to *f* = 0.15. Assuming a two-tailed alpha of.05, 31 participants per group resulted in power of 80%. Thus, the required sample size is 62 participants.

In the current study, 62 Shanghai university students were recruited (42 females, age *M* = 20.27, *SD* = 1.20). The participants were all right-handed, without physical disease, had normal or corrected-to-normal vision and had no color blindness or color weakness. Participants were also evaluated using the Chinese version of the Mini International Neuropsychiatric Interview by phone calls (32, 33). No participant was diagnosed with psychiatric disorders. The experiment was conducted between April 23, 2022, and May 7, 2022, during which Shanghai was confronted with a recurrence of the COVID-19 pandemic. All participants had been influenced by the COVID-19 lockdown measures (e.g., experiencing difficulties in buying food, or being quarantined). Information regarding the extent to which participants were affected by the COVID-19 pandemic is presented in Table 1.

**TABLE 1:** The extent to which participants were affected by the COVID-19 pandemic.

	Category	*N* (percentage)
When was the last COVID-19 case detected in your district? error bars stand for	Within 2 days	14 (22.59%)
	Between 2 days and 6 days	5 (8.06%)
	Between 7 days and 14 days	10 (16.13)
	14 days ago	33 (53.23%)
Is there a limit on your outdoor activities?	Can’t go outdoors	19 (30.65%)
	Can’t go outside the campus or the community	25 (40.32%)
	No limit	18 (29.03%)
How long have you been quarantined?	Shorter than 2 days	43 (69.35%)
	Between 2 days and 6 days	1 (1.61%)
	Between 7 days and 14 days	11 (17.74%)
	Longer than 14 days	7 (11.29%)

### 2.2. Measures

#### 2.2.1. State-trait anxiety inventory, STAI-T

Trait anxiety was assessed using the Chinese version (34) of the State-Trait Anxiety Inventory [STAI-T; (35)]. The scale consists of 20 items which are scored on a 4-point Likert scale. Higher scores indicate higher levels of trait anxiety. In the current study, the scale’s Cronbach’s α was 0.86.

#### 2.2.2. Exposure extent

Three items were created to assess the extent of exposure to the COVID-19 pandemic. The three items were: When was the last COVID-19 case detected in your district (Within 2 days = 4, Between 2 days and 6 days = 3, Between 7 days and 14 days = 2, 14 days ago = 1), Is there a limit on your outdoor activities (Can’t go outdoors = 3, Can’t go outside the campus or the community = 2, No limit = 1) and How long have you been quarantined (Shorter than 2 days = 1, Between 2 days and 6 days = 2, Between 7 days and 14 days = 3, Longer than 14 days = 4). A general score for the extent of exposure to the COVID-19 pandemic was computed by summing the three item scores.

#### 2.2.3. Experimental stimuli

The current study used word stimuli including COVID-19-related words, general threat-related words, and neutral words. COVID-19-related words were selected from the COVID-19 vocabulary approved by China International Publishing Group. General threat-related words and neutral words were taken from word lists used in previous studies examining attentional biases toward general threats (36) and the corpus of affective norms for Chinese words (37). The current study selected 10 COVID-19-related emotional words (e.g., mask, quarantine, pandemic), 10 general threat-related emotional words (e.g., violence, threat, murder), and 20 neutral words (e.g., wall, television, carpet). The used words are shown in the Appendix. All the words contained two Chinese characters and were equal in length. A neutral word was randomly paired with a COVID-19-related word or a general threat-related word to form 20 pairs of words. We recruited another 22 university students (15 females, age *M* = 20.82, *SD* = 1.10) to rate the valence and arousal of these word stimuli using a 9-point Likert scale (-4 = extremely negative or very low arousal; + 4 = extremely positive or very high arousal). There was a significant difference between the valence of COVID-19-related words (*M* = –2.69, *SD* = 0.76) and neutral words (*M* = 0.57, *SD* = 0.41), *t* (28) = 15.48, *p* < 0.001, and between general threat-related words (*M* = –2.56, *SD* = 0.57) and neutral words, *t* (28) = 17.35, *p* < 0.001, but there was no significant difference between the valence of COVID-19-related words and general threat-related words, *t* (18) = 0.43, *p* = 0.68. There was a significant difference between the arousal of COVID-19-related words (*M* = 1.84, *SD* = 0.81) and neutral words (*M* = –2.78, *SD* = 0.45), *t* (28) = 20.25, *p* < 0.001, and between general threat-related words (*M* = 1.48, *SD* = 0.75) and neutral words, *t* (28) = 19.49, *p* < 0.001, but there was no significant difference between the arousal of COVID-19-related words and general threat-related words, *t* (18) = 1.05, *p* = 0.31.

### 2.3. Procedure

**FIGURE 1:**
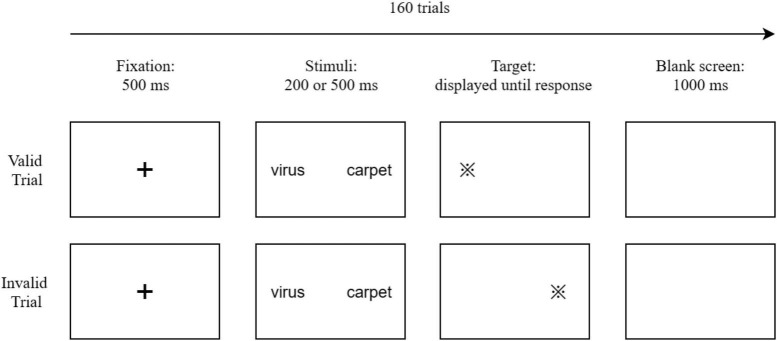
Examples of valid and invalid trials of the dot-probe task.

Due to the COVID-19 pandemic, face-to-face experiments were restricted. Thus, we conducted an online experiment. Informed consent was obtained from all participants before the start of the experiment. Participants were invited to fill out the STAI-T. Afterward, they read the instructions and completed the online experiment. The experiment was programmed in PsychoPy 2020.1.3 and was conducted on www.naodao.com. During the experiment, participants needed to share their computer screens with the researcher via an online meeting software to avoid distraction. The experiment adopted the dot-probe task. In each trial, a cross fixation “ + ” appeared in the center of the screen for 500 ms. After the fixation disappeared, one emotional word (COVID-19-related words or general threat-related words) and one neutral word were simultaneously presented on the left and the right parts of the screen. Emotional words and neutral words were presented at random positions on the left and right parts of the screen. The two words were colored white, spaced 12 cm apart, and were presented for 200 or 500 ms. After the words disappeared, a target dot (“※”) randomly appeared at one of the previous positions occupied by the two words. Participants needed to press the key “F” or “J” to judge whether “※” was on the left or the right of the screen. After participants pressed the key, the trial terminated, and the next trial commenced after a 1,000 ms blank screen (see Figure 1 for the trial structure). Before the formal experiment, the participants needed to complete 8 practice trials. Each practice trial contained two neutral words which would not appear in the formal experiment. The formal experiment contained 160 trials, and each pair of words appeared 8 times (balanced according to exposure times, positions of the emotional word, and congruent or incongruent conditions). The study protocol was approved by the University Committee on Human Research Protection of East China Normal University.

### 2.4. Data analysis

Reaction time (RT) data were analyzed after removing incorrect responses (3%). Median RTs were used to reduce the effect of outliers in the dot-probe task (38, 39). For each participant, we calculated the attentional bias index for each stimuli type (COVID-19 and general threat) at both exposure times (200 ms and 500 ms) by subtracting the median RT in congruent trials when the probe appeared at the position of the threat from the median RT in incongruent trials when the probe appeared at the position of the neutral stimuli, according to Mogg et al. (40). Positive values of attentional bias index indicate vigilance toward threat-related words, while negative values indicate avoidance away from threat-related words. All statistics were computed using IBM SPSS Statistics 26.0.

## 3. Results

### 3.1. Descriptive statistics and correlations

The median STAI-T score was 43.5 (*M* = 45.03, *SD* = 8.40). In the current study, participants who had an STAI-T score below 43.5 were assigned to the low anxiety group (*N* = 31, 21 females, age *M* = 20.06, *SD* = 1.03) and participants who had an STAI-T score above 43.5 were assigned to the high anxiety group (*N* = 31, 21 females, age *M* = 20.48, *SD* = 1.34). The independent samples *t*-test showed there was a significant difference in the scores on STAI-T between the low anxiety group (*M* = 38.23, *SD* = 3.96) and the high anxiety group (*M* = 51.84, *SD* = 5.66), *t* (60) = 10.97, *p* < 0.001.

All participants had an accuracy rate above 80% (*M* = 97%, *SD* = 0.03). We adopted an exclusion criterion on error rates above 20% used by Fani et al. (30). Thus, no participant was excluded from the analysis. The mean RTs in each condition for the dot-probe task are displayed in Table 2. Correlations among the study variables are displayed in Table 3.

**TABLE 2:** The mean RTs (ms) for each condition in the dot-probe task (*SD*s in parentheses).

		Low trait anxiety		High trait anxiety	
Exposure time	Stimuli type	Congruent	Incongruent	Congruent	Incongruent
200 ms	Virus	397.57 (41.73)	396.57 (37.40)	401.57 (61.00)	408.77 (69.92)
	General threat	394.45 (33.16)	398.49 (46.26)	421.23 (106.06)	402.57 (62.32)
500 ms	Virus	419.41 (82.98)	403.75 (51.08)	416.08 (92.60)	429.34 (135.22)
	General threat	411.54 (76.15)	406.60 (54.33)	427.10 (108.95)	431.11 (94.74)

**TABLE 3:** Correlations among the study variables.

	1	2	3	4	5
1. Trait anxiety	–				
2. Exposure extent	–0.108	–			
3. Attentional bias index for COVID-19-related words under 200 ms	0.046	0.017	–		
4. Attentional bias index for COVID-19-related words under 500 ms	0.132	0.010	0.108	–	
5. Attentional bias index for general threat-related words under 200 ms	–0.157	0.051	–0.152	–0.479^**^	–
6. Attentional bias index for general threat-related words under 500 ms	–0.039	–0.186	–0.369^**^	0.286*	–0.369^**^

*p < 0.05, **p < 0.01.

### 3.2. Analysis of variance

We conducted a 2 × 2 × 2 mixed design analysis of variance (ANOVA) on attentional bias index with Anxiety Group (low and high) as a between-participant factor and Exposure Time (200 ms and 500 ms) and Stimuli Type (COVID-19 and general threat) as within-participant factors. Anxiety Group had no main effect on the attentional bias index, *F*(1, 60) = 1.68, *p* = 0.20, η_p_^2^ = 0.03. Exposure Time had no main effect on the attentional bias index, *F* (1, 60) = 0.02, *p* = 0.90, η_p_^2^ = 0.00. Stimuli Type had no main effect on attentional bias index, *F* (1, 60) = 0.54, *p* = 0.47, η_p_^2^ = 0.01.

**FIGURE 2:**
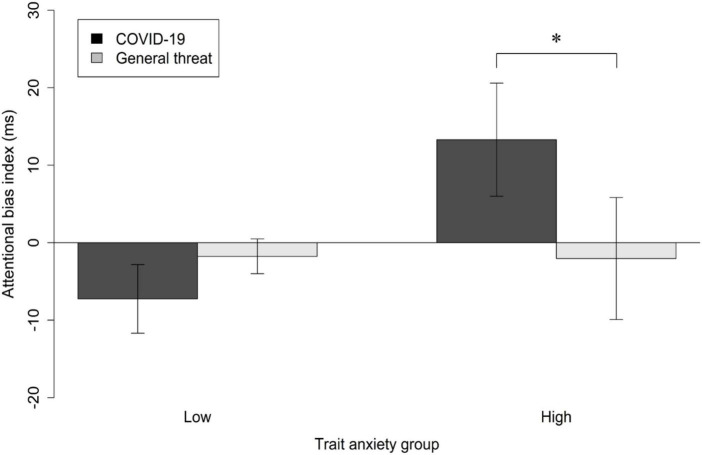
Attentional bias index (ms) for COVID-19-related and general threat-related words in the low and high anxiety groups. The error bars stand for Standard Errors.

There was a significant Anxiety Group × Stimuli Type interaction on the attentional bias index (see Figure 2), *F* (1, 60) = 4.40, *p* = 0.040, η_p_^2^ = 0.07. Further simple effect analysis of the interaction revealed that participants in the high anxiety group paid more attention to COVID-19-related words (*M* = 10.23, *SD* = 34.37) than general threat-related words (*M* = –7.33, *SD* = 31.26), *p* = 0.050. For participants in the low anxiety group, there was no significant difference between COVID-19-related words (*M* = –8.91, *SD* = 33.73) and general threat-related words (*M* = –0.46, *SD* = 21.69), *p* = 0.349.

There was no significant Anxiety Group × Exposure Time interaction, *F* (1, 60) = 3.09, *p* = 0.08, η_p_^2^ = 0.05. There was no significant Exposure Time × Stimuli Type interaction, *F* (1, 60) = 1.42, *p* = 0.24, η_p_^2^ = 0.02. There was no significant three-way Anxiety Group × Exposure Time × Stimuli Type interaction, *F* (1, 60) = 0.25, *p* = 0.62, η_p_^2^ = 0.00.

## 4. Discussion

The present study is the first to examine the differences between attentional bias toward COVID-19-related and general threat-related stimuli in trait anxiety. Participants with high trait anxiety exhibited an attentional bias toward COVID-19-related stimuli and an attentional bias away from general threat-related stimuli, while participants with low trait anxiety showed an attentional bias away from both types of stimuli. The results indicate that individuals with high trait anxiety are more susceptible to chronic stress exposure and show specific attentional bias toward threatening information corresponding to their current worries.

We found the attentional bias toward COVID-19-related stimuli among individuals with high trait anxiety. This result aligned with previous studies which found a positive association between attentional bias toward COVID-19 stimuli and health anxiety (3) and COVID-19 anxiety syndrome (8). The COVID-19 pandemic could be regarded as a traumatic stressor (1). After experiencing traumas, individuals may develop pathological cognitive structures and are prone to interpret mild stimuli as threatening, especially those stimuli which are similar to their previous traumatic experiences (41). Thus, they are likely to exhibit excessive behavioral and psychological responses to these stimuli (30). However, these stimuli may be only moderately threatening or even neutral for individuals without corresponding trauma exposure. The susceptibility to trauma exposure is more pronounced in individuals with high trait anxiety due to their emotion dysregulation and severer stress responses (42, 43). Under repetitive exposure to COVID-19-related negative information, individuals with high trait anxiety may be more prone to develop a pathological learning pattern. They could learn from their previous negative experiences to fear COVID-19-related stimuli, show elevated sensitivity to potential COVID-19 threats, and allocate more attention to COVID-19-related stimuli. Although this selective attention allocation may have been adaptive during the initial stages of the COVID-19 pandemic, the persistence of such cognitive patterns may impede information processing and prolong anxiety symptoms (30). Thus, it is important to focus on individuals’ mental health after a mass stressor such as the COVID-19 pandemic or natural disasters and provide psychological intervention.

Notably, we did not find an attentional bias toward general threat-related stimuli among individuals with high trait anxiety. This result was inconsistent with a previous meta-analysis which indicated a stable pattern of anxiety-linked attentional bias toward threats (4). The inconsistency could be explained by content specificity in anxiety-linked attentional bias. A meta-analysis revealed there was a greater attentional bias toward disorder-congruent threatening stimuli than disorder-incongruent threatening stimuli (9). For example, Foa et al. (44) found among rape victims that trauma-related words elicited a stronger attentional bias than other threat-related words. Stefan et al. (45) found that among individuals with illness anxiety disorder, the disengagement bias was stronger for health-related stimuli than general threat-related stimuli. These results suggest that attentional bias is most significant when threatening stimuli correspond to an individual’s current worries (46). According to several cognitive models, previous memory and learning could play a role in schema-driven threat processing (4, 47). During the COVID-19 pandemic, the perceived possibility of encountering general threats may have been low, while the worry of contamination and quarantine had become the core challenge. Although both COVID-19-related threats and general threats are biologically significant, the burden caused by COVID-19 threats is more pervasive and prolonged. Unlike short-term stress exposure, long-term stress exposure can result in severer physiological and behavioral dysregulation (48). Therefore, under chronic stress exposure to the COVID-19 pandemic, individuals with high trait anxiety may have learned to specifically fear COVID-19-related information, leading to corresponding attentional bias. Pergamin-Hight et al. (9) suggested future studies on attentional bias move beyond disorder-congruent content specificity to explore personalized specificity in more generalized disorders rather than disorders with a specific concern. The current study contributes to the field by exploring the relationship between trait anxiety and content specificity related to participants’ personal experiences.

We did not find an attentional bias toward either COVID-19-related or general threat-related stimuli among individuals with low trait anxiety. This result is consistent with previous studies (4). Although it has been suggested that individuals with low trait anxiety also selectively attend to threats, the threshold of threat intensity required to elicit such an effect is higher (49). It is plausible that the word stimuli used in our study did not possess sufficient valence or “threat value” to induce an attentional bias in individuals with low trait anxiety. In contrast to word stimuli, picture stimuli possess higher emotional salience (50) and may provoke stronger emotional reactions and attentional biases. We did not find significant association between exposure extent and attentional bias indexes. Previous research suggested trauma exposure may contribute to attentional bias via the activation of fear structure (51). The COVID-19 pandemic is characterized as pervasive and persistent, but less intense than common traumatic events (e.g., bereavement, natural disasters). It may be possible that the intensity of the COVID-19 pandemic as a stressor is not adequate to activate the fear structure. Moreover, the current study only measured objective exposure to the COVID-19 pandemic. Considering the heterogeneous psychological outcomes after the COVID-19 pandemic (1), subjective trauma exposure may have a stronger association with attentional bias.

About the components of attentional bias, we did not find a significant effect of exposure time on the attentional bias index. Participants with high trait anxiety showed attentional bias toward COVID-19-related stimuli and then maintained their attention, within the time course from 200 ms to 500 ms. In contrast, they at first biased away from general threat-related stimuli and then directed their attention to general threat-related stimuli. Participants with low trait anxiety showed an opposite pattern. These results align with a meta-analysis that found attentional bias in a wide range of exposure times among anxious individuals (4). However, it remains unclear whether the positive values of the attentional bias index are attributed to vigilance toward threats or delayed disengagement from threats. To address the limitation of the traditional attentional bias index, Koster et al. (52) introduced a variant version of the dot-probe task involving neutral-neutral stimuli pairs and found only delayed disengagement. Additionally, as indicated by Cisler and Koster (53), the components of attentional bias, mediating mechanisms (e.g., attentional control, emotion regulation goal), and stages of information processing may interact with each other. Thus, future research should use paradigms that separate vigilance from delayed disengagement and consider integrating mediating factors.

The current study has implications for increasing intervention efficacy, particularly for ABMT and post-disaster interventions. Prior meta-analyses examining ABMT have demonstrated small-to-medium effect sizes for reducing anxiety symptoms (54, 55). To improve the therapeutic effect, the nature of training stimuli needs to be considered (9). Given that attentional bias is influenced by personal experiences and concerns, a one-size-fits-all approach may not be effective in treating anxiety symptoms. An optimized intervention procedure could incorporate personalized content-specific threat stimuli, as the attentional bias toward such stimuli is stronger and may have a more significant impact on anxiety symptom maintenance than general threat-related information. Additionally, following a mass stressor, such as the COVID-19 pandemic or natural disasters, individuals with high trait anxiety are more prone to developing a pathological fear structure unique to their traumatic experiences, thus increasing the risk of anxiety disorders. Therefore, a timely intervention targeted at this vulnerable group is imperative to prevent maladaptive post-trauma responses and overgeneralized fear.

Despite the valuable contributions of the present study, certain limitations should be acknowledged. First, the sample size of the current study is only 62, which may be the reason why there is no significant correlation between trait anxiety and attentional bias. Previous studies which found a significant correlation between trait anxiety scores and attentional bias indexes had a larger sample size. For example, Salemink et al.’s study (2007) recruited 133 participants and Rudaizky et al.’s study (2014) recruited 72 participants. A larger sample size is needed to explore the linear relationship between trait anxiety and attentional bias. Second, the cross-sectional design precluded the ability to establish causal relationships between trait anxiety and attentional bias. To address this limitation, future investigations may consider implementing a longitudinal design to explore whether a bidirectional and mutually facilitating causality exists between anxiety and attentional bias (6). Additionally, the university student sample may not be representative enough of clinical samples. A Chinese sample with generalized anxiety disorder has a mean score on trait anxiety of 54.82 (56), whereas the high trait anxiety group in the current study has a mean score of 51.84. The extent of trait anxiety in the current study may not be adequate to elicit a strong effect of attentional bias. Thus, it is recommended that future research replicate the present findings among clinical samples with generalized anxiety disorders to determine the generalizability of the conclusions. Moreover, it is important to note that the traditional attentional bias index did not differentiate between vigilance toward threats and delayed disengagement from threats and response bias may have influenced the RT-based dot-probe task (29). To overcome these limitations, novel experimental methods with higher psychometric properties and moment-to-moment dynamic characteristics, such as eye-tracking and event-related potentials, are suggested for measuring specific cognitive processing stages (57, 58).

## 5. Conclusion

The present study contributes to the field by providing evidence on content-specific attentional bias toward COVID-19-related stimuli in trait anxiety. Participants with high trait anxiety exhibited an attentional bias toward COVID-19-related stimuli and attentional bias away from general threat-related stimuli, while participants with low trait anxiety showed attentional bias away from both types of stimuli. Our findings suggest several theoretical implications. We contribute to the field of content specificity by investigating personalized specificity in a group of individuals who shared similar chronic stress exposure. The current study also has clinical implications for ABMT and post-disaster intervention. The use of content-specific stimuli which are related to participants’ personal experiences could improve intervention efficacy.

## Data availability statement

The raw data supporting the conclusions of this article will be made available by the authors, without undue reservation.

## Ethics statement

The studies involving humans were approved by the University Committee on Human Research Protection of East China Normal University. The studies were conducted in accordance with the local legislation and institutional requirements. The participants provided their written informed consent to participate in this study.

## Author contribution

YZ: Data curation, Writing−original draft. XJ: Investigation, Methodology, Writing−review and editing. SP: Software, Visualization, Writing−review and editing. HJ: Writing−review and editing. YW: Conceptualization, Funding acquisition, Methodology, Project administration, Resources, Supervision, Writing−review and editing.

## Appendix

Neutral words: 短语, 参数, 章节, 地毯, 广播, 胶卷, 食谱, 电视, 服装, 营地, 蜡烛, 墙壁, 期刊, 抽纸, 冰箱, 橱窗, 火车, 大厅, 飞机, 贴纸 (English version: phrase, parameter, chapter, carpet, broadcast, film, recipe, television, clothes, camp, candle, wall, journal, tissue, refrigerator, shopwindow, train, hall, plane, sticker).

COVID-19-related words: 口罩, 病毒, 传染, 采样, 发热, 肺炎, 隔离, 确诊, 密接, 疫情 (English version: mask, virus, contagion, nucleic acid test, fever, pneumonia, quarantine, diagnosis, close contact, pandemic).

General threat-related words: 暴力, 危险, 威胁, 谋杀, 攻击, 子弹, 杀害, 粗暴, 腐烂, 毒蛇 (English version: violence, danger, threat, murder, attack, bullet, kill, cruel, rot, snake).
